# Integrated multi-omics analyses identify key anti-viral host factors and pathways controlling SARS-CoV-2 infection

**DOI:** 10.21203/rs.3.rs-1910932/v1

**Published:** 2022-08-15

**Authors:** Jiakai Hou, Yanjun Wei, Jing Zou, Roshni Jaffery, Shaoheng Liang, Caishang Zheng, Ken Chen, Pei-Yong Shi, Yiwen Chen, Xuping Xie, Weiyi Peng

**Affiliations:** University of Houston; The University of Texas MD Anderson Cancer Center; University of Texas Medical Branch; University of Houston; The University of Texas MD Anderson Cancer Center; The University of Texas MD Anderson Cancer Center; The University of Texas MD Anderson Cancer Center; UTMB; The University of Texas MD Anderson Cancer Center; University of Texas Medical Branch; University of Houston

**Keywords:** Genome-wide CRISPR Screen, SARS-CoV-2, host factors

## Abstract

Host anti-viral factors are essential for controlling SARS-CoV-2 infection but remain largely unknown due to the biases of previous large-scale studies toward pro-viral host factors. To fill in this knowledge gap, we performed a genome-wide CRISPR dropout screen and integrated analyses of the multi-omics data of the CRISPR screen, genome-wide association studies, single-cell RNA-seq, and host-virus proteins or protein/RNA interactome. This study has uncovered many host factors that were missed by previous studies, including the components of V-ATPases, ESCRT, and N-glycosylation pathways that modulated viral entry and/or replication. The cohesin complex was also identified as a novel anti-viral pathway, suggesting an important role of three-dimensional chromatin organization in mediating host-viral interaction. Furthermore, we discovered an anti-viral regulator KLF5, a transcriptional factor involved in sphingolipid metabolism, which was up-regulated and harbored genetic variations linked to the COVID-19 patients with severe symptoms. Our results provide a resource for understanding the host anti-viral network during SARS-CoV-2 infection and may help develop new countermeasure strategies.

## Introduction

The 2019 coronavirus disease (COVID-19) pandemic, caused by severe acute respiratory syndrome coronavirus 2 (SARS-CoV-2), has already claimed over six million lives and resulted in global economic disruption^1^. As a newly emerged coronavirus, SARS-CoV-2 is an enveloped, single-stranded, positive-sense RNA virus^2^. Similar to other coronaviruses, SARS-CoV-2 hijacks a broad range of host factors to complete its infection cycle, including viral entry, replication, virion assembly, and dissemination. Although COVID-19 vaccines have successfully prevented severe disease and death^3–5^, SARS-CoV-2 variants with improved transmissibility and immune evasion continue to emerge, leading to the prolonged pandemic and breakthrough infections. A better understanding of virus-host interactions will provide new strategies for countermeasure development.

Unbiased virus-host interactome screens and genetic screens have advanced our understanding of SARS-CoV-2 biology. In virus-host interactome screens, cellular proteins that interact with viral proteins are pulled down by affinity purification (AP) using tagged viral proteins, followed by mass spectrometry (MS) to determine the identities of the interacting proteins. This approach has identified hundreds of SARS-CoV-2 interacting proteins that are involved in epigenetic regulation, mRNA translation machinery, protein post-translational modifications, and innate immune responses^6,7^. Some of the viral interacting proteins, such as Sigma receptors, display *in vitro* anti-viral activity^8^. In genetic screens, the impact of CRISPR guide RNA (gRNA)-mediated perturbation of an individual host gene in response to viral infection-mediated cytopathic effects (CPEs) is evaluated. Genes whose expression significantly affects CPEs are identified as pro-viral or anti-viral host factors. For SARS-CoV-2, both loss-of-function (LOF) and gain-of-function (GOF) screens have been performed by using the CRISPR knockout (KO) system^9–17^ and the CRISPR activation system^16,18,19^. These studies confirm the prominent roles of several known host factors in SARS-CoV-2 infection, such as ACE2 and TMEM30A as receptors to bind viral spike proteins, and TMPRSS2 and cathepsin L as key proteases for viral entry. More importantly, these screens provide a wealth of information on novel pro-viral host factors including KCNA6, TMEM41B, THEM106B, HMGB1, class III PI3K subunits, and proteins in the SWI/SNF chromatin remodeling complex.

However, recently reported CRISPR genetic screens are largely biased for identifying pro-viral host factors for SARS-CoV-2. Despite genome-wide, bidirectional CRISPR KO screens have been conducted by only two independent groups^16,17^, all loss-of-function screens were under the condition of low multiplicities of infection (MOI) (range from 0.01-1) of virus for a long period of selection (ranging from 7-14 days post-infection). Under this condition, host cells are selected by many rounds of viral infection cycles, which could bias to identify host factors that regulate cell growth under the infection condition. Another weakness of this long selection screen is the loss of high complexities of the gRNA library in the pooled samples at the interrogation time point(s), when viral selection lasts more than 7 days. As gRNAs targeting potential pro-viral factors should be enriched in pooled samples, the library complexity in the pooled sample is not critical for the identification of these factors. However, keeping high library complexity is important to capture anti-viral host factors, whose gRNAs should be underrepresented in pooled samples.

Here, we performed a genome-wide CRISPR dropout screen using a 2-day SARS-CoV-2 infection to better identify physiologically relevant anti-viral host factors. The identified host factors were analyzed for their clinical relevance by integrating databases of virus-host interactome, genome wide association analysis (GWAS), and single-cell transcriptome of COVID-19 patients. The top 30 novel hits (4 of pro-viral factors and 26 of anti-viral factors) were individually validated for their phenotypes observed in our genome-wide dropout screen. Among the validated hits, we characterized the role of two pro-viral factors (ATP6V0D1 and DPAGT1) and three anti-viral factors (DAZAP2, VTA1, and KLF5) in regulating viral replication for mechanistic insights. Taken together, our study has broadened the understanding of the virus-host interaction during SARS-CoV-2 infection and has identified host risk factors associated with COVID-19 severity.

## Results

### Genome-wide CRISPR dropout screens identify host factors controlling vulnerability to SARS-CoV-2 infection.

**Figure 1:**
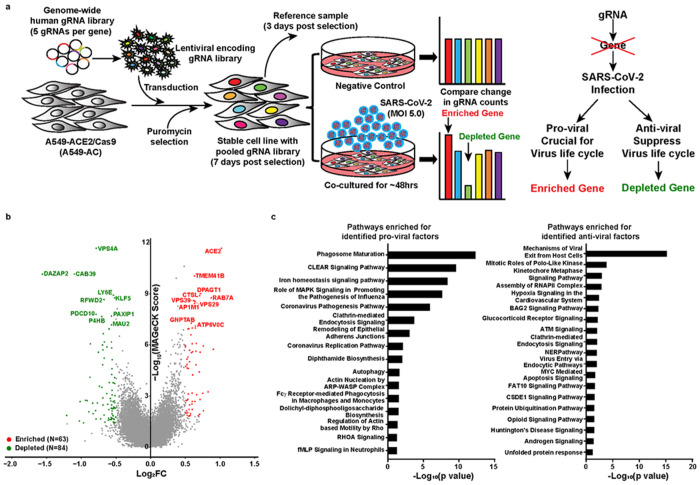
Discovery of host factors controlling SARS-CoV-2 infection. (a) A schematic diagram of the functional CRISPR/Cas9 dropout screen based on virus-induced cytopathic effect (CPE). A549-AC cells were transduced with a genome-wide human gRNA library (five sgRNAs per gene) and followed by puromycin selection. After 3-day puromycin selection, 30 million pooled cells were collected as the reference sample. On day 7 after selection, pooled A549-AC cells were infected with recombinant SARS-CoV-2 at MOI=5 for 48 hours. Pooled A549-AC cells without viral treatment were severed as the controls. The changes in gRNA distribution between the viral infected samples and controls were determined. (b) A volcano plot showing top candidates for pro-viral and anti-viral host factors. The gene-level MAGeCK scores and the changes of gRNA distribution between A549-AC cells with and without viral infection were calculated. The log2 fold change of the second-best gRNA for each gene was selected for data representation. Genes whose gRNAs were significantly enriched or depleted in the infected group (p value <0.05 and |log_2_FC| ≥0.5) were labeled as red and green dots, respectively. The top ten enriched/depleted (pro-viral/anti-viral) genes based on MAGeCK scores were indicated. (c) Ingenuity pathway analysis of identified host factors for SARS-CoV-2 infection. Enriched pathways for pro-viral factors (enriched, left panel) and anti-viral factors (depleted, right panel) with statistical significance (p value< 0.05) were illustrated.

We performed a genome-wide LOF screen based on virus-induced cytopathic effect. Our LOF screen was optimized to identify depleted hits with potential anti-viral activities. This screen employed a SARS-CoV-2-permissive lung epithelial cell line, A549-AC, which constitutively expresses human ACE2 and Cas-9 (Extended Data Fig. 1a and 1b). Cells with gRNAs targeting anti-viral host factors would be significantly depleted after SARS-CoV-2 infection, whereas those with gRNAs targeting pro-viral host factors would be enriched (Fig. 1a). To achieve 50-80% of cell killing (an optimal selection condition for dropout screens)^20^, we infected A549-AC cells with different multiplicity of infection (MOI, range from 0.1 to 40) and measured cell viability at 48-hour (h) post-infection. The results showed an MOI of 4.24 to yield 50% cell killing at 48-h post-infection (Extended Data Fig. 1c). Thus, the MOI of 5 was selected in our screen. Indeed, at 48 hours post-infection, around 50% of the cells exhibited CPE (Extended Data Fig. 1d). Based on genomic DNA amount isolated from the control and infected groups, our dropout screen reached a 47% of cell killing rate. Our screen exhibited sufficient gRNA reads for all sequenced samples, thus maintaining the library complexity (over 10 million reads from 30 million cells per sample; Extended Data Fig. 2a). The frequencies of gRNAs among triplicate samples are highly correlated, demonstrating the consistency of our barcode sequencing results (Extended Data Fig. 2b). A proportion of gRNAs in the essential gene group was depleted after the 7-day expansion, when compared with the non-essential gene groups (Extended Data Fig. 2c). However, the gRNAs targeting essential genes was not depleted in the reference sample. The results confirmed that, prior to SARS-COV-2 infection, the 7-day expansion had successfully removed gRNAs targeting genes involved in cell proliferation and survival (Extended Data Fig. 2c).

Next, we determined the fold change (FC) in gRNA frequencies in cells with and without SARS-CoV-2 infection and evaluated the impact of each genetic perturbation using the Model-based Analysis of Genome-wide CRISPR/Cas9 Knockout (MAGeCK)^21^. Figure 1b summarizes the top ten enriched (pro-viral factors) and depleted (anti-viral factors) genes. Among them, three pro-viral hits (*ACE2, THEM41B* and *CTSL*) and one anti-viral hit (*LY6E*) were in common with previously published reports^13,14,16^. Using an FC cut-off in gRNA frequency (|Log_2_FC|≥0.5) and a statistically significant threshold of *p*<0.05, we found 63 enriched and 84 depleted genes and named them as pro-viral hits and anti-viral hits, respectively (Extended Data Tables 1 and 2). Gene ontology (GO) analysis showed the pro-viral hits are not only from known signaling pathways required for viral replication and pathogenesis, but also from the pathways involved in phagosome maturation, endocytosis, and autophagy (Fig. 1c). In contrast, top anti-viral hits are from pathways involved in viral entry and exit, and cellular RNA polymerase II complex assembly (Fig. 1c). The functions of many identified pathways, such as iron homeostasis signaling, DNA repair, and protein ubiquitination, remain to be determined (Fig. 1c).

### Integrative analyses reveal clinical relevance of identified host factors in COVID-19 patients.

**Figure 2:**
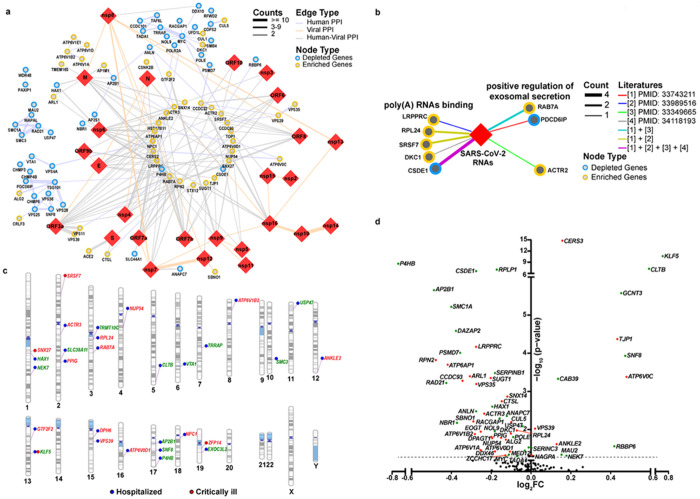
Integrative analysis revealing virus-host interactome networks and potential clinical relevance of identified host factors. (a) Protein-protein interactome (PPI) networks between viral proteins and host factors identified by the dropout screen. 229 interactions between 26 SARS-CoV-2 proteins (red diamonds) and 147 human proteins (circles; depleted hits: blue; enriched hits: yellow) were found. The color of edge indicates the type of interaction (purple: host-host PPI; orange: viral-viral PPI; grey: host-viral PPI) and the thickness of edge indicates the count number of published datasets. (b) RNA-protein interactome networks between the viral RNA and host factors identified by the dropout screen. SARS-CoV-2 viral RNA was indicated as the red diamond; identified host factors were represented as circles (enriched hits: yellow; depleted hits: blue). The interaction between RNA and identified host factors were indicated as different edge types (colors: literature; thickness: count number of published manuscripts). (c) Gene variations in multiple identified host factors are associated with disease severity in COVID-19 patients. The Genome-Wide Association Study (GWAS) between variants of identified host factors and clinical features was performed by using the COVID19-hgGWAS meta-analyses. “Hospitalized” indicates that single nucleotide polymorphisms (SNPs) of the identified host factors were related to the hospitalized COVID-19 patients, which were labeled with blue dots. “Critically-ill” indicates SNPs of the identified host factors were related to COVID-19 patients with particularly severe respiratory symptoms, which were labeled with red dots. The names of enriched genes and depleted genes were labeled in red and green, respectively. (d) A volcano plot showing the changes of mRNA expression of identified host factors in epithelial cells from COVID-19 patients with and without severe illness. Multiple scRNA-seq datasets obtained from cells in bronchoalveolar lavage fluid of COVID-19 patients were extracted. COVID-19 patients with mild symptoms or hospitalized in the ward were stratified in the mild group, whereas COVID-19 patients with severe symptoms or hospitalized in the intensive care unit (ICU) were stratified in the severe group. The fold change of gene expression and p value were calculated. Identified pro-viral factors and anti-viral factors which are differentially expressed (p<0.05) in these two groups were highlighted with red and green dots, respectively.

To shed light on the potential mechanisms of host factors in SARS-CoV-2 infection, we first constructed a protein-protein interaction (PPI) network that contained 114,366 PPIs covering 13,716 human proteins and 30 SARS-CoV-2 proteins, based on the published PPI datasets^22^. We then constructed a sub-network that only covered 147 CRISPR screen hits (63 enriched and 84 depleted) and 26 SARS-CoV-2 proteins, including 79 PPIs between CRISPR screen hits, 22 PPIs between SARS-CoV-2 proteins, and 128 PPIs between SARS-CoV-2 and CRISPR hits (Fig. 2a). As shown in Figure 2a, there are three major host PPI sub-networks. The first anti-viral sub-network is composed of the subunits of cohesin complex, including SMC1 A, SMC3, RAD21, and the cohesin complex release factor WAPL. Cohesin complex plays a key role in regulating three-dimensional (3D) chromatin organization^23^. The second PPI sub-network is from the components of the ESCRT (The endosomal sorting complexes required for transport) pathway, including CFIMP4B, TSG101, VPS28, SNF8, CFIMP6, VTA1, VPS25, and PDCD6IP. The ESCRT pathway has been shown to play an important role in regulating the infection of enveloped viruses, such as HIV^24^. The discovery of ESCRT-related PPI sub-network as anti-viral factors suggests that ESCRT pathway may also play an important function in mediating SARS-CoV-2 infection. The third PPI sub-network is related to c-MYC and its interacting proteins, such as TRRAP. TRRAP was originally known as a co-activator for c-MYC and is critical for its oncogenic activities^25^, suggesting an involvement of c-MYC-mediated transcriptional regulation in SARS-CoV-2 infection. Additionally, the host PPI sub-network analysis identified subunits of vacuolar-type ATPase (V-ATPase; including ATP6V1 El, ATP6V1D, ATP6V1B2, and ATP6V1 A) as pro-viral factors (Fig. 2a).

We also constructed an RNA-protein interaction (RPI) network between SARS-CoV-2 RNAs and host proteins. This network includes 452 nodes (The SARS-CoV-2 RNAs are represented by one node) and 706 edges. The CRISPR screen hits that formed an interaction with SARS-CoV-2 RNAs fall into two major categories (Fig. 2b). The first category is the poly(A)-binding proteins, including four pro-viral factors (LRPPRC, RPL24, SRSF7, and DKC1) and one anti-viral factor (CSDE1). RPL24 was recently reported to be involved in RNA metabolism, such as translation and splicing^26^. LRPPRC encodes an RNA-binding protein (RBP) that binds to poly(A)-mRNAs in the nucleus and mitochondria; LRPPRC is required for human immunodeficiency virus (HIV)-1^27^ and hepatitis C virus (HCV) infections^28^. CSDE1 is an RBP that participates in the regulation of translation and mRNA turnover and is required for coxsackievirus B3 (CVB3) infection^29^. The second category includes the proteins that are positive regulators of exosomal secretion, including two pro-viral factors (RAB7A, PDCD6IP) and one anti-viral factor (ACTR2; Fig. 2b).

To uncover the clinical relevance of identified host factors, we integrated our CRISPR screen data with the COVID-19 GWAS meta-analysis results. The analysis showed that gene variations in 29 identified host factors (15 for pro-viral and 14 for anti-viral factors) were significantly associated with the critically ill and/or hospitalized COVID-19 patients (*p*<0.001; Fig. 2c). In accordance with the PPI analyses, we found that the V-ATPase subunit ATP6V1B2, the c-Myc co-activator TRRAR and the cohesin complex subunit SMC3 were associated with the COVID-19 patient hospitalization, suggesting their clinical relevance to disease. We also found that KLF5, a transcription factor of the Krüppel-like factor subfamily of zinc finger proteins that showed anti-viral activity in our CRISPR screen, was associated with COVID-19 hospitalization and severe symptoms (Fig. 2c).

Lastly, we extracted transcriptional expression results of identified hits in epithelial cells from published scRNA-seq datasets of airway cells in COVID-19 patients^9^ and compared differences between mild COVID-19 patients and severe COVID-19 patients. Our results showed that 59 of 147 identified hits displayed differential expression between these two groups (p<0.05; Fig. 2d). Based on the principle that pro-viral factors and anti-viral factors are expected to be upregulated and downregulated in severe COVID-19 patients, respectively, the concordance of changes of differentially expressed genes (DEG) between CRISPR screen and scRNA-seq data was examined. We observed 24 out of 30 identified pro-viral factors, and 8 out of 29 anti-viral factors showed a concordant up- or down-regulation in the severe COVID-19 patients compared with the mild ones (Fig. 2c), suggesting these pro- and anti-viral factors may contribute to the severity of viral pathogenesis. Interestingly, KLF5 (anti-viral factor) displays the most dramatic upregulation in mild patients when compared with severe patients (Fig. 2c). Together with the GWAS analysis, these results highlight the clinical relevance of KLF5 as a novel anti-viral factor.

### Assessment of novelty and individual validation of hits selected by the CRISPR dropout screens

**Figure 3:**
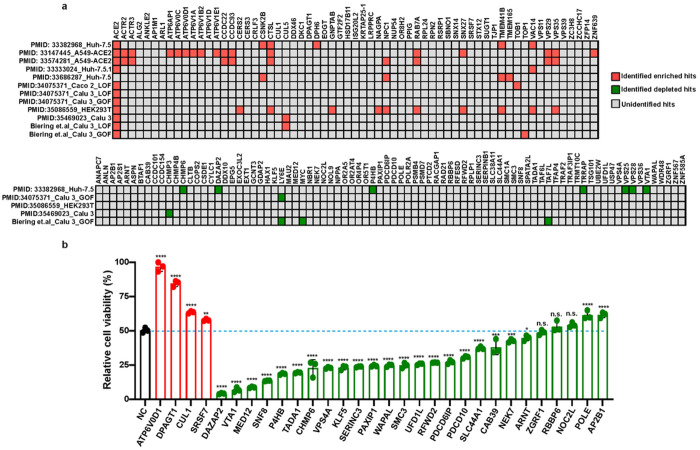
Validation of host factors identified from the CRISPR dropout screen. (a) Performance of identified host factors in previously reported SARS-CoV-2 screens. For each dataset, enriched and depleted hits that meet the listed criteria were marked as red and green squares, respectively. Others that fail to be identified in the listed datasets were marked as gray squares. (b) Effects of perturbation of top 30 hits on the CPE caused by SARS-CoV-2 infection. Four pro-viral and twenty-six anti-viral factors were selected for validation. A series of A549-AC cell lines expressing gene-specific gRNAs were infected with recombinant SARS-CoV-2 at MOI= 2.5. The percentages of viable cells were measured at 48 hours post-infection. Data were normalized using the viability of corresponding cells at mock condition. Statistical significance between cells expressing gene-specific gRNAs and non-targeting gRNA (NC) was determined by one-way ANOVA with repeated measurements. At least two independent experiments were performed, and samples were triplicated in each independent experiment. *p<0.05; **p<0.01; ***p<0.001; ****p<0.0001. n.s., not significant.

We compared the hits from our screen with those from previous SARS-CoV-2 screens. Extended data Table 3 summarizes the 12 datasets from genome-wide CRISPR screens based on the CPE of SARS-CoV-2 on human epithelial cells. Although the majority of these screens were enrichment screens and focused on the discovery of pro-viral host factors, 5 of them included evaluation of depleted hits (anti-viral factors). Based on publicly available results, we set different criteria to determine whether our selected candidates are marked as putative host factors in corresponding screens (Extended data Table 3). Among the hits selected by our screen, 29 enriched (46%) and 11 depleted (13%) hits can be identified by at least one of the listed datasets (Fig. 3a). Well-recognized pro-viral host factors such as ACE2, CTSL, and TMEM41B were identified by our screens and at least three additional CRISPR screens, validating the power of this genetic approach in exploring pro-viral factors in SARS-CoV-2 infection. However, significant amounts of the hits, particularly depleted hits, from our CRISPR dropout screens using 48-h infection time were not identified in previous studies. These results underscore the importance of our CRISPR dropout screens in filling in the knowledge gap of anti-viral host factors in SARS-CoV-2 infection.

Next, we selected 4 novel pro-viral factors and 26 top-ranking anti-viral factors for individual validation. For each of the 30 selected genes, we prepared gRNA-expressing gene-knockout A549-AC lines. Two independent sets of infection experiments were performed at the MOI of 2.5 and 5. The A549-AC line expressing non-targeting gRNAs served as a control line in all validation experiments. The results showed that 66.7% of the selected screen hits were validated in both sets of independent experiments (Extended data Table 4). As shown in one representative experiment at MOI of 2.5 (Fig. 3b), inhibition of all tested 4 pro-viral factors significantly reduced the CPE after SARS-CoV-2 infection, with the most reduction observed in the *ATP6V0D1* and *DPAGT1* KO lines. On the other hand, knocking out 21 of 26 tested anti-viral factors increased the cell death of infected cells, with the most increase observed in the *DAZAP2* and *VTA1 KO* lines (Fig. 3b). Similar perturbation effects were observed at the condition with MOI of 5 (Extended data Fig. 3a). Although multiple tested hits are also implied to directly control the growth of A549 cells (Extended data Fig. 3b), the impact of each perturbation on cell growth does not correlate with their effect on susceptibility to CPE of infected cells. Furthermore, we used the cell number of each perturbation without viral infection to normalize the CPE in our validation experiments. Thus, these results implied that hits identified from our dropout screens are not biased to genes that regulate cell growth. Taken together, literature cross-checks and individual validation demonstrate that our dropout screens successfully revealed a set of novel host factors/pathways with essential roles in SARS-CoV-2 infection.

### Mechanistic insights of top-ranking host factors in determining viral entry, attachment, and replication.

Based on the phenotype of each perturbation in individual validation experiments, we focused on two pro-viral factors (ATP6V0D1 and DPAGT1) and two anti-viral factors (DAZAP2 and VTA1) whose knockout resulted in the most dramatic changes in the CPE of infected cells for further mechanistic studies. KLF5, one of the top 10 anti-viral factors, was also selected because of its clinical relevance as indicated by both GWAS analysis (Fig. 2c) and transcriptome analysis of COVID-19 patients (Fig. 2d). The expression of *KLF5* in epithelial cells from severe COVID-19 patients was significantly lower than that from mild COVID-19 patients (Extended data Fig. 4). This trend was not observed when we compared *KLF5* expression between non-COVID-19 pneumonia patients with variable severity, suggesting a specific role of KLF5 as an anti-viral factor in modulating disease severity of COVID-19 patients. The expressions of pro-viral *ATP6V0D1* and *DPAGT1* and anti-viral Z74Z4P2were upregulated, whereas no difference in the expression was observed for pro-viral *VTA1*, in severe COVID-19 patients (Extended data Fig. 4).

**Figure 4:**
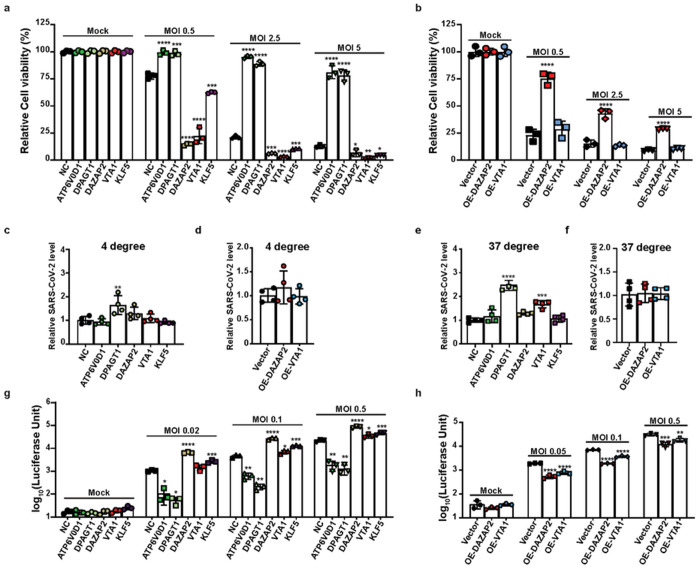
Impacts of top-ranking host factors on virion entry and replication pathways. (a-b) Effects of perturbation of top-ranking host factors on CPE caused by SARS-CoV-2 infection at different infection conditions. For gene-specific knockdown (KD) effect (a), two pro-viral factors (*ATP6V0D1, DPAGTT*) and three anti-viral factors (*DAZAP2, VTA1, KLF5*) were selected. For gene-specific overexpression effect (b), two anti-viral factors (*DAZAP2, VTA1*) were selected. Genetically modified A549-AC cells were infected with recombinant SARS-CoV-2 at MOI=0.5, 2.5, and 5 for 48 hours. A549-AC cells expressing a non-targeting gRNA (NC) or the GFP vector were served as control cells for the KD and overexpression experiments, respectively. Data were normalized using the viability of corresponding cells at mock condition. (c-f) Effects of perturbation of top-ranking host factors on SARS-CoV-2 attachment and entry. Genetically modified A549-AC cells were infected with recombinant SARS-CoV-2 at MOI =1. To evaluate the changes in the viral attachment (c and d), the infection was performed at 4°C for 1 h; whereas to evaluate the changes in viral entry (e and f), the infection was performed at 37°C for 1 h. The levels of RNAs encoding the viral N protein and *ACTB* mRNAs were determined by RT-PCR. Viral RNA levels were normalized using the expression of *ACTB* mRNA. (g-h) Effects of perturbation of top-ranking host factors on SARS-CoV-2 replication. A549-AC cells with gene-specific KD (g) or overexpression (h) were infected with SARS-CoV-2-Nluc at MOI= 0.02, 0.1, and 0.5. The luciferase signals were measured at 24 hours post-infection. Statistical significances between cells with gene-specific overexpression and control cells at each infection condition were determined by one-way ANOVA with repeated measurements. At least two independent experiments were performed, and samples were triplicated in each independent experiment. *p<0.05; **p<0.01; ***p<0.001; ****p<0.0001. n.s., not significant.

We explored the function of the five selected hits in SARS-CoV-2 infection. We first confirmed the knockout of each gene in the A549-AC cell lines by real-time PCR and/or Western blot (Extended data Fig. 5a and. 5b). Infection of these gRNA-expressing A549-AC lines with different MOIs of SARS-CoV-2 produced consistent phenotypes: (i) knockout pro-viral ATP6V0D1 or DPAGT1 increased cell viability and (ii) knockout anti-viral DAZAP2, VTA1, or KLF5 decreased cell viability (Fig. 4a). Next, we attempted to produce overexpressing cell lines for individual genes. Among the five selected genes, we only successfully generated stable A549-AC lines exogenously expressing *DAZAP2 or VTA1* (Extended data Fig. 5c and 5d). Interestingly, only overexpression of DAZAP2 (DAZAP2-OE) increased the cell viability of SARS-CoV-2-infected cells, whereas overexpression of *VTA1* (VTA1-OE) did not affect the cell viability of infected cells (Fig. 4b).

We examined the steps of SARS-CoV-2 infection that are modulated by the identified host factors. To access virus attachment to cells with altered expression of selected factors, we incubated the KO or OE cells with SARS-CoV-2 at 4°C for 1 h (which allowed the virus to attach to cells without entry) and measured the virions attached to the cell surface by viral RNA-based real-time PCR. Only DPAGT1-KO cells showed moderately enhanced viral attachment, but not in other types of KO or OE cells (Figs. 4c and 4d). We also co-cultured these cells with viral particles at 37°C for 1 h (which allows virus attachment and entry into cells) to determine the impact of each perturbation on virus entry. Consistently, DPAGT1-KO and, to a less extent, VTA1-KO increased virus entry. However, no significant changes were observed in other cell lines when compared with control cells (Fig. 4e and 4f). These results suggest that only DPAGT1 facilitates virus attachment and/or entry.

Finally, we compared viral replication of SARS-CoV-2 expressing nanoluciferase (SARS-CoV-2-Nluc) on different knockout and overexpression cell lines. We previously showed that luciferase activity can be reliably used to quantify viral replication^30^. Despite variable magnitude, we observed a significant decrease in viral replication in ATP6VOD1-KO and DPAGT1-KO cells, and an increase in replication in DAZAP2-KO, VTA1-KO, and KLF5-KO cells. Conversely, DAZAP2-OE and VTA1-OE cells showed decreased viral replication, further confirming their anti-viral effect. Altogether, our results indicate that DPAGT1 facilitates SARS-CoV-2 attachment and entry, whereas ATP6V0D1, DAZAP2, VTA1, and KLF5 affect post-entry stages of SARS-CoV-2 replication.

## Discussion

We performed the first genome-wide dropout screens using 48-h SARS-CoV-2 infection, and integrated our screen results with GWAS analysis, viral RNA and protein interactomes, and transcriptional profiles of epithelial cells in COVID-19 patients. Despite our experimental condition being optimized to identify anti-viral host factors of SARS-CoV-2, several well-recognized pro-viral host factors/pathways, including ACE2, CTSL, and the autophagy pathway, were re-validated in our screens. Additionally, 25 of the 30 hits identified from our screens were confirmed by individual gene validation. These results indicate the reproducibility of our screen and provide confidence in the list of putative host factors generated in this study. Importantly, when compared with the results from published genomic SRAS-CoV-2 screens (either enrichment screens or bi-direction screens), a significant portion of the host factors in our list (87% of anti-viral factors and 54% of pro-viral factors) were not identified by previous screen conditions, suggesting the importance of our dropout screens in using 48-h infection time to identify novel anti-viral factors.

By leveraging viral RNA and protein interactomes, we found that six anti-viral factors identified in this study belong to the cohesin complex, a central organizer of the three-dimensional (3D) genome structure^31^. Consistent with our finding, a recent study showed that SARS-CoV-2 was able to rewire host chromatin organization in human cells to confer immunological gene deregulation^32^. Our finding of the subunits of the cohesin complex as inhibitors of SARS-CoV-2 infection provides the first functional evidence to directly support the role of 3D chromatin organization in mediating host-virus interactions.

We selected five novel host factors for mechanistic studies. *ATP6V0D1* encodes a transmembrane V0 domain D subunit of Vacuolar-type H+-ATPase (V-ATPase), which is the primary proton pump for H+ homeostasis and mediates acidification of eukaryotic intracellular organelles. Besides ATP6V0D1, we found a group of V-ATPases interact with the M protein of SARS-CoV-2. These proteins are highly conserved enzymes and are often localized to the plasma membranes of many cell types^33^. Plasma membrane V-ATPases play crucial roles in pH homeostasis and coupled transport^34,35^. Perturbing *ATP6V0D1* in host cells has limited impacts on viral attachment and entry of SARS-CoV-2. However, silencing *ATP6V0D1* reduces viral replication and decreases CPE. *DPAGT1* is another novel pro-viral host factor selected for further studies. It encodes an enzyme that catalyzes the initial step of protein N-glycosylation^36^. N-glycosylation of asparagine is a common post-translational modification, which has been reported to regulate protein stability and function^37^. In our study, knockout *DPAGT1* promotes viral entry, but reduces viral replication. Furthermore, higher expression of these two pro-viral factors was found in severe COVID-19 patients. These results suggest the role of V-ATPases and N-glycosylation in the pathogenesis of COVID-19.

DAZAP2 was identified as an anti-viral factor that contains several Src homology (SH) domains. As an adaptor protein, DAZAP2 plays important roles in regulating several important biological and pathological processes such as DNA damage^38^, inflammation^39^, and activation of Wnt signaling^40^. Although there is no interaction between DAZAP2 and SARS-CoV-2 proteins or RNAs in our interactomes analysis, DAZAP2 was identified as a flavivirus RNA-binding host factor by using yeast three-hybrid systems^41^. Knockout and overexpression of *DAZAP2* significantly increased and decreased viral replication, respectively; however, neither perturbation affected virus entry. These results provide a strong rationale to determine whether *DAZAP2* interacts with viral RNAs/proteins and, if so, how these interactions modulate viral replication.

VTA1 is a component from ESCRT, which sorts membrane proteins for degradation in the lysosomes. The ESCRT-related PPI sub-network was found in the interactome analysis of our anti-viral hits. Increased viral entry was observed in *VTA1* KO cells, suggesting its potential role in viral internalization. More interestingly, we observed strong correlations between COVID-19 severity and *KLF5* expression. The variants in *KLFT* genomic region and reduced *KLF5* expression are associated with severe COVID-19 patients. KLF5 belongs to a highly conserved Krüppel-like zinc finger transcription factor family, which plays crucial roles in various physiological and pathological processes, including cell cycle, angiogenesis, migration, apoptosis, autophagy inflammation, self-renew, and differentiation^42^. KLF5 was recently recognized as a transcriptional orchestrator of epidermal cell differentiation and pathophysiology of ischemic heart failure by governing sphingolipid metabolism^43–45^. As glycosphingolipid is essential for viral replication, its expression level is significantly induced upon SARS-CoV-2 infection^46^. These results implied that KLF5 could regulate SARS-CoV-2 replication by modulating sphingolipid metabolism. This notion is further supported by the clinical observation that reduced sphingosine levels are associated with the development of symptomatic COVID-19 patients compared to asymptomatic counterparts^47^.

In summary, our genetic dropout screen and integrative database analysis reveal a broad range of novel molecules and pathways including the cohesin complex, V-ATPases, N-glycosylation, and sphingolipid metabolism, all of which modulate viral replication and/or COVID-19 manifestation. As the function of these host factors is largely unknown, our findings have broadened the understanding of the pathogenesis of COVID-19, particularly about anti-viral host factors.

## Methods

### Cell lines and recombinant SARS-CoV-2

A549 and FIEK293T cell lines were obtained from the American Type Culture Collection (ATCC, Bethesda, MD). All cell lines and their genetically modified cell lines were cultured in Dulbecco’s modified Eagle’s medium (DMEM) supplemented with 10% heated-inactivated fetal bovine serum (FBS; #S11150, R&D System, Minneapolis, MN) and 100μg/ml of Normocin (ant-nr-1, InvivoGen, San Diego, CA). The A549-hACE2 cells that stably express human *ACE2* were generated previously^30^ and grown in the culture medium supplemented with 10 μg/mL Blasticidin (#A1113903; ThermoFisher, Waltham, MA). Cells were grown at 37 °C with 5% CO2. All cell lines were authenticated by short tandem repeat fingerprinting or the expression of tagged markers used for genetic modification. The mycoplasma detection kit (#13100-01, SouthernBiotech, Birmingham, AL) was used to routinely monitor for mycoplasma contamination of cultured cells. The maximum length of time of *in vitro* cell culture between thawing and use in the described experiments was two weeks.

The recombinant SARS-CoV-2 was generated by a previously described reverse genetic system based on the strain 2019-nCoV/USA_WA1/2020 derived from the first patient diagnosed in the US^48^. The recombinant SARS-CoV-2 was used to screen host factors regulating CPE. The nanoluciferase severe respiratory syndrome coronavirus 2 (SARS-CoV-2-Nluc) established in our previous study^30^ was used to evaluate the involvement of identified host factors in viral entry and replication. Experiments with SARS-CoV-2 and nano-Luciferase virion were performed in a BSL-3 laboratory by personnel equipped with powered air-purifying respirators. All procedures were followed by biosafety protocols approved by the Institutional Biosafety Committees at the University of Houston and the University of Texas Medical Branch at Galveston.

### Establishment of genetically modified cell lines

Lentivirus-based gene delivery was used to either genetically knockout gene-of-interests (GOIs) or ectopically express GOI. To generate lentiviral supernatants, HEK293T cells were seeded 16 hours prior to transfection and transfected with the lentiviral vectors encoding different gRNAs or GOIs, along with lentiviral packaging plasmids, pCMV-VSV-G and psPAX2 (#8454 and #12260, Addgene) by the jetPRIME transfection reagent (#101000046, VWR, Radnor, PA) according to the manufacturer’s protocol. Viral supernatants were collected 72 hours post-transfection and filtered by 0.45 mm PVDF Syringe Filter Unit (#SLHV033NK, Millipore-Sigma, Burlington, MA) to remove cell debris. Designated titers of lentivirus were used to infect cells in the presence of 8μg/ml hexadimethrine bromide (#107689, Sigma-Aldrich, St Louis, MO).

To generate A549 cells expressing Cas-9 for gene editing, A549-hACE2 cells were transduced lentivirus expressing lentiCas9-EGFP (#63592, Addgene). The Cas-9 expressing A549-hACE2 (A549-AC) line was generated by sorting GFP^+^ cells 72 hours after lentivirus transduction. To genetically suppress the expression of GOIs in cells, lentiviral gRNA-expressing vectors were constructed. Fully synthesized double strand (ds) DNA fragments (Twist Bioscience, San Francisco, CA) encoding gene-specific gRNAs were inserted into the lentiGuide-Puro (#52963, Addgene) as previously described^20^. The forward sequences of dsDNA fragments were listed in the extended data table 5. In addition, a vector encoding non-targetable gRNA was also constructed to generate control cell lines. A549-AC cells were transduced with newly constructed gRNA-expressing vectors and followed by antibiotic selection using 1μg/ml of puromycin (#A1113803, Gibco, Carlsbad, CA) to generate stable cell lines with knocking out of GOIs. Cells transduced with the viral vector encoding a non-targetable gRNA were generated and served as control cells. To ectopically express GOIs in cells, dsDNA fragments encoding Flag-tagged human open reading frames (ORFs) of *DAZAP2* (NM_014764.4) and *VTA1* (NM_016485.5) were inserted into the lentiviral vector, pLVX-IRES-ZsGreen1 (#PT4064-5, Takara Bio, San Jose, CA). A549-hACE2 cells were transduced with lentiviral ORF vectors. Stable cell lines were generated by sorting GFP^+^ cells 72 hours after lentivirus transduction. Cells transduced with pLVX-IRES-ZsGreen1 were generated and served as control cells.

### CRISPR dropout screens

The optimized human genome-wide knockout (KO) CRISPR Library (H1) consisting of 92,817 gRNAs targeting 18,436 genes (5 gRNAs for each gene) was purchased from Addgene (Pooled Library #1000000132) and used to generate lentivirus as described above. 1.5×10^8^ of A549-AC cells were transduced with pooled library lentivirus at a low multiplicity of infection (MOI; ~0.15-0.2) to ensure that each cell would receive only one gRNA as previously described^20^. 48 hours after transduction, cells were cultured in the growth medium in the presence of 1 mg/ml puromycin to select transduced cells. 72 hours after puromycin selection, 30 million of cells were collected and used as the reference sample. 7 days post puromycin selection, pooled gRNA-expressing A549-AC cells were re-seeded in T175 cell culture flasks (10 million/flask). The next day, for the group receiving virus infection, SARS-CoV-2 was used to infect 2×10^8^ of pooled A549-AC cells at MOI =5 for 48 hours. Pooled A549-AC cells cultured in the same assay medium were collected and served as the control group. 48 hours post SARS-CoV-2 infection, non-adhesive cells were removed by repeated wash using pre-warmed PBS. Adherent cells were harvested by using 0.25% Trypsin-EDTA (#15050065, ThermoFisher) and washed three times using PBS. Three replicated samples were collected for each group.

Genomic DNA from all cell samples was extracted by using TRIzol (#15596026; ThermoFisher) according to the manufacturer’s protocol. DNA fragments containing gRNA sequences were amplified and barcoded with adaptation by nested polymerase chain reaction (PCR) as previously described^20^. The quality and concentration of all PCR products were determined by the Qubit ssDNA high sensitivity assay kit (#Q10212; ThermoFisher) and the bioanalyzer High Sensitivity DNA Kit (#5067-4626 2100; Agilent, Santa Clara, CA) respectively. Samples were then sequenced by Illumina Next-Generation Sequencing (NSG) at the MD Anderson Cancer Center Advanced Technology Genomics Core.

### Assays to evaluate the virus-induced cytopathic effect

A series of genetically modified A549-AC cell lines (10,000 cells per well in DMEM medium containing 2% FBS) were plated into clear flat-bottom 96-well plates. On the next day, the recombinant SARS-CoV-2 was used to infect pre-seeded A549-AC cells at designated MOIs (0.5, 2.5 or 5). 48 hours after viral infection, 4 μL of Cell Counting Kit-8 (#CCK-8, Sigma-Aldrich, St Louis, MO) was added to each well. After incubation at 37 °C for 90 min, absorbance at 450 nm was measured using a Cytation5 multi-mode microplate reader (BioTek, Winooski, VT). The relative cell viability was calculated by normalizing the absorbance of the control groups (set as 100%). At least two independent experiments were performed to determine the sensitivity of genetically modified cells to virus-induced cytopathic effect. For each experiment, triplication was performed for all groups.

### Assays to evaluate viral entry and replication

A series of genetically modified A549-AC cell lines (10,000 cells per well in DMEM medium containing 2% FBS) were plated into were seeded in white opaque 96-well plates. On the next day, the recombinant SARS-CoV-2-Nluc virus was used to infect pre-seeded A549-AC cells at designated MOIs (0.02, 0.1, and 0.5). 18 hours after infection, cells were applied to Nano-Glo® Dual-Luciferase® reporter assays (#N1610; Promega, Madison, Wl) according to the manufacturer’s instructions. Luciferase signals from all samples were measured using a Synergy™ Neo2 microplate reader. At least two independent experiments were performed to determine the sensitivity of genetically modified cells to virus-induced cytopathic effect. For each experiment, triplication was performed for all groups.

### Assays to evaluate viral entry and attachment

A series of genetically modified A549-AC cell lines (25,000 cells per well in DMEM medium containing 2% FBS) were plated into were seeded in 12-well plates. On the next day, SARS-CoV-2 was used to infect pre-seeded cells at MOI=1.0. For the virus entry assay, cells were co-incubated with SARS-CoV-2 at 37°C for 1 h. For the viral attachment, cells were co-incubated with the virus at 4°C for 1 h. After co-incubation, RNAs were isolated from infected cells by Trizol and Direct-zol RNA Miniprep Kits (#R2050; Zymo Research, Irvine, CA) according to the manufacturer’s instructions. The relative expression levels of the SARS-CoV-2 nucleocapsid (N) protein were determined from the quantitative Real-time PCR (qRT-PCR) using the iTaq Universal One-Step RT-qPCR Kit (#1725151; Bio-Rad, Flercules, CA). Triplication of PCR reactions was included in all assays. The expression levels of *ACTB* were used for data normalization. The sequences of RT-PCR primers are listed in the extended data table 6.

### Immunoblot analysis

To verify the expression of GOIs, proteins were extracted by lysed cells using RIPA Lysis and Extraction Buffer (#89900; ThermoFisher) and the concentrations of protein samples were quantified with the Pierce BOA Protein Assay Kit (#23225; ThermoFisher). The western blot analysis was used to determine the expression of protein-of-interest. The intensity of protein bands was detected by Immobilon Western Chemiluminescent HRP Substrate (#WBKLS0500; MilliporeSigma, Burlington, MA) using the ChemiDoc Imaging System. The antibody targeting b-actin (8H10D10, #3700) was purchased from the Cell Signaling Technology (Danvers, MA), human ACE2 (AF933) was purchased from R&D Systems, DAZAP2 (G-4, sc-515182) was purchased from Santa Cruz Biotechnology (Dallas, TX), KLF5 (21017-1-AP) was purchased from Proteintech Group (Rosemont, IL) and the monoclonal ANTI-FLAG antibody (M2, #F3165) was purchased from MilliporeSigma.

### Bioinformatic analysis

The MAGeCK (vO.5.9.4) count module was used to calculate the read count of individual sgRNAs in different samples with the following parameters: “-l human_sgrna_sequences_A.library --control-sgrna human_sgrna_sequences_A.library.negctrl --norm-method control --sample-label C-1,C-2,C-3,control 1,control2,control3,Ref-1 -n COVID-19_CRISPR_210212.count --fastq files.fq”. MAGeCKtest module was then applied with parameters “-k COVID-19_CRISPR_210212.count.txt-c control 1,control2,control3-t C-1,C-2,C-3-norm-method control --keep-tmp -n COVID-19_CRISPR_210212_C_control --control-sgrna human_sgrna_sequences_A.library.negctrl --gene-lfc-method secondbest”, to identify the genes that showed a significant differential selection (|log_2_(fold-change)| > 0.5 and *p<* 0.05) between the control and SARS-CoV-2 infection groups.

### GWAS and interactome analysis

The COVID-19 GWAS meta-analyses results (release 6)^49^ for “Hospitalized covid vs. population” and “Very severe respiratory confirmed covid vs. population” were downloaded from the COVID19 Host Genetics Initiative (https://www.covid19hg.org/). The CRISPR screen hits that are within +/−10kb of the SNPs reaching the significance level of *p*< 0.001 in the GWAS meta-analysis were then identified as candidate genes associated with “Hospitalized” and “Critically-ill” conditions.

We also performed an integrated analysis of host-host and host-viral interactome, based on the protein-protein interaction (PPI) data from the BioGRID database (Release 4.4.205)^22^. Briefly, we first constructed a human-human and human-SARS-CoV-2 PPI (CoV-2_HsPPI) network that contained the human-human or human-viral PPIs supported by at least two independent experiments which resulted in a total of 114,366 interactions covering 13,716 human proteins and 30 SARS-CoV-2 proteins. By selecting the CRISPR screen hits and the SARS-CoV-2 proteins as the seed nodes, a sub-network was then constructed as the CRISPR screen hits related host-host and host-viral PPI network.

Besides the PPI, we integrated four host-viral protein-RNA interactome (RPI) datasets^50–53^ that characterize the interaction between human protein and SARS-CoV-2 RNAsto construct a, “SARS-CoV-2 RNAs – human proteins” (CoV-2_HsRPI) network that included 452 nodes and 706 edges. By selecting the CRISPR screen hits as the seed nodes, a sub-network was then constructed as the CRISPR screen hits related RPI network.

### scRNAseq analysis

scRNA-seq data of airway epithelial cells were extracted from the dataset generated by Wauters *et al*^54^, which include 65,166 cells from 35 pneumonia patients, 22 of whom tested positive for SARS-CoV-2, while the other 13 are infected by other pathogens. Among all the patients, 14 of whom experienced mild symptoms, while the other 21 are severe cases. The data analysis was performed as described in previous studies by Baggen et al^9^. Briefly, based on the source of cells, epithelial cells were dissected into four groups, namely non COVID19_mild, non COVID19_severe, COVID19_mild, and COVID19_severe. To test the hypothesis that whether candidates identified in our screen are differentially expressed in among epithelial cells derived from different groups, we use the Kruskal–Wallis test to identify if any group of cells are significantly different from another, and if so, the Wilcoxon rank-sum test was used as a post-hoc test to identify the pairs of groups that are significantly different.

### Statistical analyses

Summary statistics (e.g., mean, SEM) of the data are reported. Assessments of differences in continuous measurements between two groups were made using two-sample t-test. Multiple group comparisons were performed by Analysis of Variance (ANOVA) with repeated measures. A p-value of less than 0.05 was considered significant. Graph generation statistical analyses were performed using the Prism software program (GraphPad Software), Tableau 8.2 software program (Tableau Software), and R software programming language (version 3.1.0). The sample size for each experiment was chosen based on the study’s feasibility given its exploratory nature.

## Supplementary Material

1

2

3

4

5

6

7

## Data Availability

Raw data files from genome-wide CRISPR drop-off screen are accessible from GEO (GSE209750).
