# Supplementary material for: Integrated multi-omics analyses identify key anti-viral host factors and pathways controlling SARS-CoV-2 infection
Source: Res Sq. 2022 Aug 15:rs.3.rs-1910932. Preprint. [Version 1] doi: 10.21203/rs.3.rs-1910932/v1 (PMC9413708; doi:10.21203/rs.3.rs-1910932/v1)
Supplement: 1 [file 20220726ExtendedDataFiguresandFigurelegends.pdf]

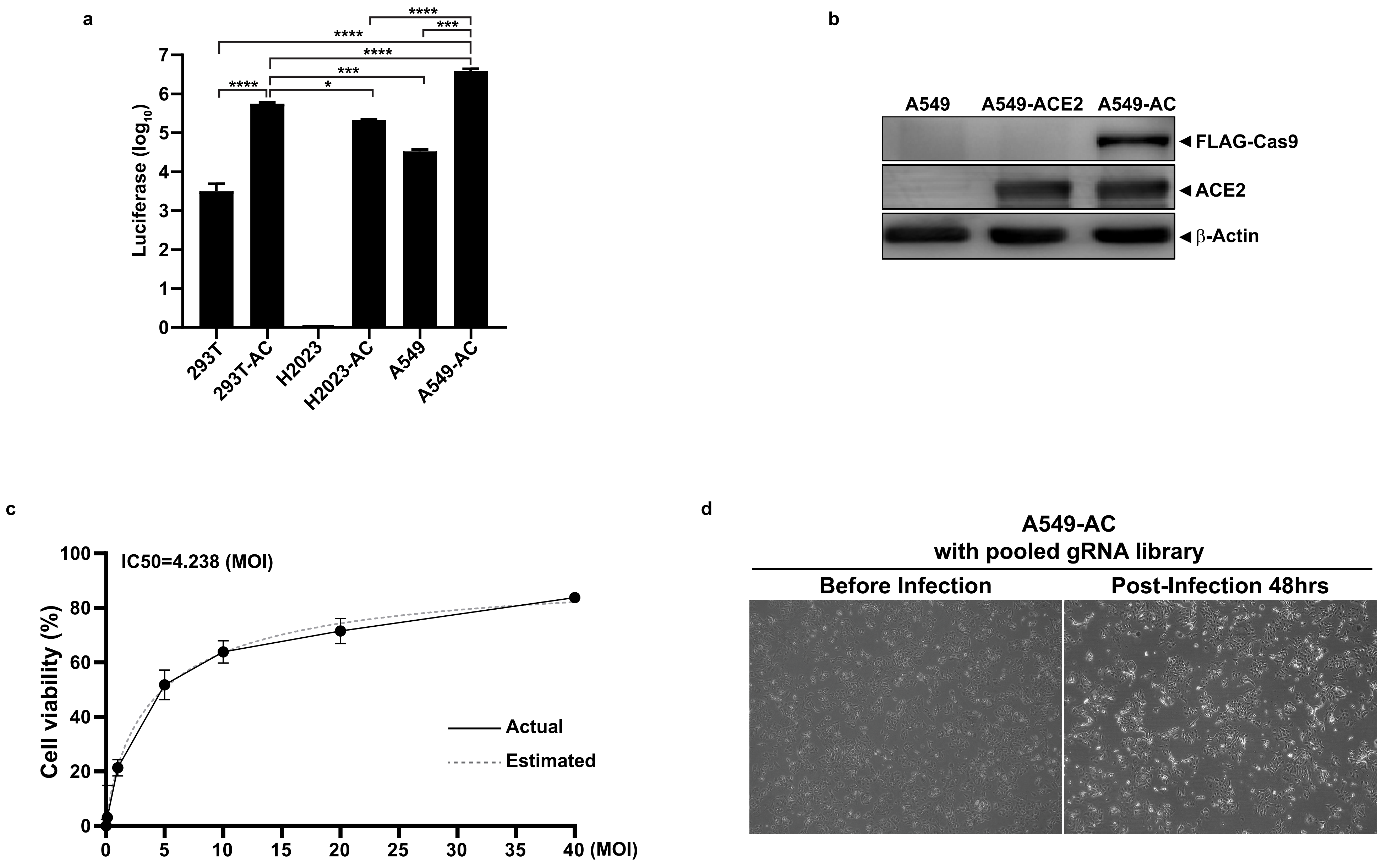
a
Luciferase (log10)
0
1
2
3
4
5
6
7
293T
293T-AC
H2023
H2023-AC
A549
A549-AC
****
***
****
****
***
*
****
b
A549
A549-ACE2
A549-AC
◄FLAG-Cas9
◄ACE2
◄β-Actin
c
IC50=4.238 (MOI)
Cell viability (%)
0
20
40
60
80
100
0
5
10
15
20
25
30
35
40 (MOI)
Actual
Estimated
d
A549-AC
with pooled gRNA library
Before Infection
Post-Infection 48hrs

**Extended Data Figure 1. Establishment of genome-wide CRISPR/Cas9 dropout screens for SARS-CoV-2 infection** (a) Evaluation of permissiveness of human epithelial cell lines for SARS-CoV-2 infection. Human epithelial cells were infected with recombinant SARS-CoV-2-Nluc at MOI=0.2 and luciferase signals were measured at 24-h post-infection. (b) Expression of ACE2 and Cas9 expression in A549-AC cells. Anti-human ACE2 and anti-flag antibodies were used to determine the level of ACE and FLAG-tagged Cas9, respectively. (c) Dose effect of SARS-CoV-2 on CPE. A549-AC cells were infected with different MOIs (ranging from 0.1 to 40) of recombinant SARS-CoV-2. 48 hours after infection, the viabilities of infected cells were measured. A four-parameter nonlinear regression method was used to generate the estimated dose-response curve and to calculate the MOI for 50% of cell lysis. (d) Representative images of cells before and after viral infection. Typical bright field images of pooled A549-AC cells with the gRNA library were illustrated before (left panel) and after (right panel) SARS-CoV-2 infection. Samples were triplicated in experiments. The comparisons with statistical significance were indicated. *$p<0.05$; ***$p<0.001$; ****$p<0.0001$.

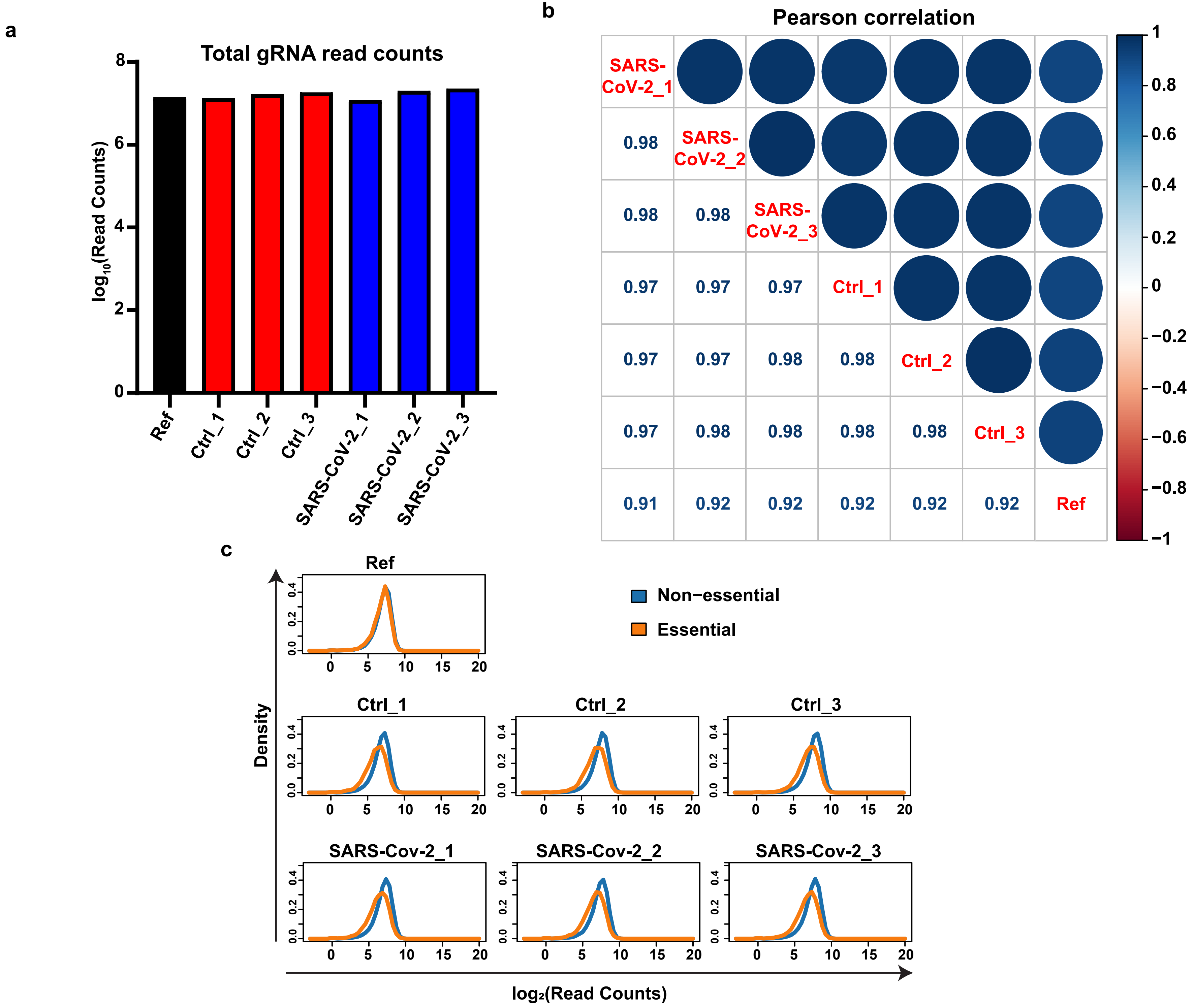
a
Total gRNA read counts
log10(Read Counts)
8
6
4
2
0
Ref
Ctrl_1
Ctrl_2
Ctrl_3
SARS-CoV-2_1
SARS-CoV-2_2
SARS-CoV-2_3
b
Pearson correlation
SARS-CoV-2_1
SARS-CoV-2_2
SARS-CoV-2_3
Ctrl_1
Ctrl_2
Ctrl_3
Ref
0.98
0.98 0.98
0.97 0.97 0.97
0.97 0.97 0.98 0.98
0.97 0.98 0.98 0.98 0.98
0.91 0.92 0.92 0.92 0.92 0.92
1
0.8
0.6
0.4
0.2
0
−0.2
−0.4
−0.6
−0.8
−1
c
Ref
Ctrl_1
Ctrl_2
Ctrl_3
SARS-Cov-2_1
SARS-Cov-2_2
SARS-Cov-2_3
Non−essential
Essential
Density
log2(Read Counts)
0.0
0.2
0.4
0
5
10
15
20

**Extended Data Figure 2. Quality evaluation of results from the genome-wide CRISPR dropout screen.** (a) Raw read counts of total gRNAs in samples collected from the genome-wide CRISPR dropout screen. (b) Correlations of gRNA frequencies across experimental samples. Pairwise Pearson correlation analyses were performed between two distinct samples. (c) Distribution of gRNAs targeting essential and non-essential genes in the reference sample (upper panel), the control samples (middle panel) and the SARS-CoV-2 infected samples (bottom panel).

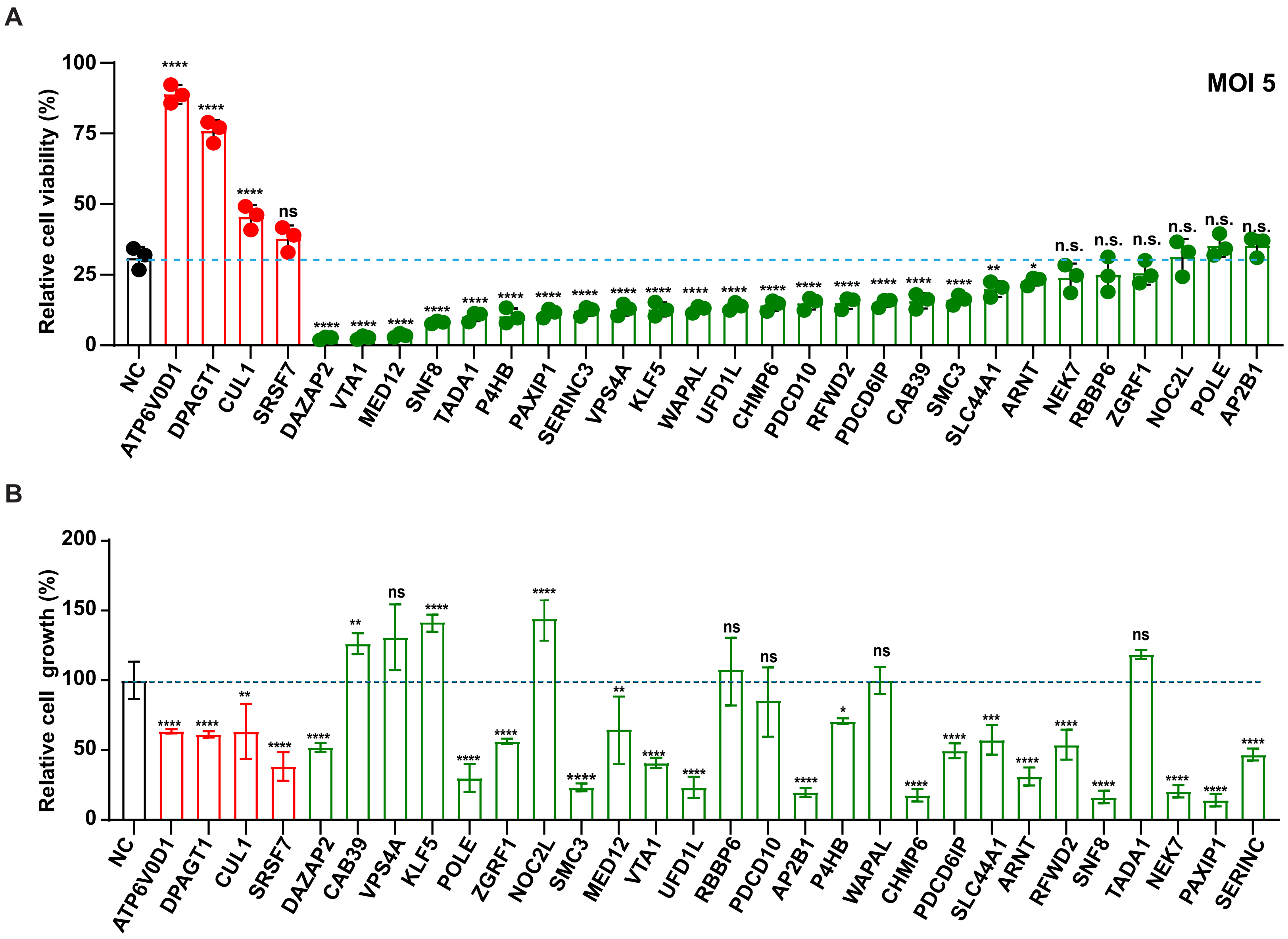
A
MOI 5
Relative cell viability (%)
100
75
50
25
0
NC
ATP6V0D1
DPAGT1
CUL1
SRSF7
DAZAP2
VTA1
MED12
SNF8
TADA1
P4HB
PAXIP1
SERINC3
VPS4A
KLF5
WAPAL
UFD1L
CHMP6
PDCD10
RFWD2
PDCD6IP
CAB39
SMC3
SLC44A1
ARNT
NEK7
RBBP6
ZGRF1
NOC2L
POLE
AP2B1
B
Relative cell growth (%)
200
150
100
50
0
NC
ATP6V0D1
DPAGT1
CUL1
SRSF7
DAZAP2
CAB39
VPS4A
KLF5
POLE
ZGRF1
NOC2L
SMC3
MED12
VTA1
UFD1L
RBBP6
PDCD10
AP2B1
P4HB
WAPAL
CHMP6
PDCD6IP
SLC44A1
ARNT
RFWD2
SNF8
TADA1
NEK7
PAXIP1
SERINC

**Extended Data Fig. 3. Phenotypes of knocking out putative host factors in lung epithelial cells.** (a) Effects of knocking out putative host factors on CPE at the high MOI condition. A series of A549-AC cell lines with related gRNA expression were infected with the recombinant SARS-CoV-2 at MOI=5 and cell viability was measured at 48 hours post-infection. (b) Effects of knocking out putative host factors on *in vitro* growth. Equal numbers of genetically modified A549-AC cells were seeded and cultured for 48 hours *in vitro*. The relative changes in cell numbers of A549-AC cells with gene-specific KD were calculated by normalizing with the number of A549-AC cells expressing non-targeting gRNA. Statistical significance between the gRNAs and NC was determined by one-way ANOVA with repeated measurements. *$p<0.05$; **$p<0.01$; ***$p<0.001$; ****$p<0.0001$. n.s., not significant.

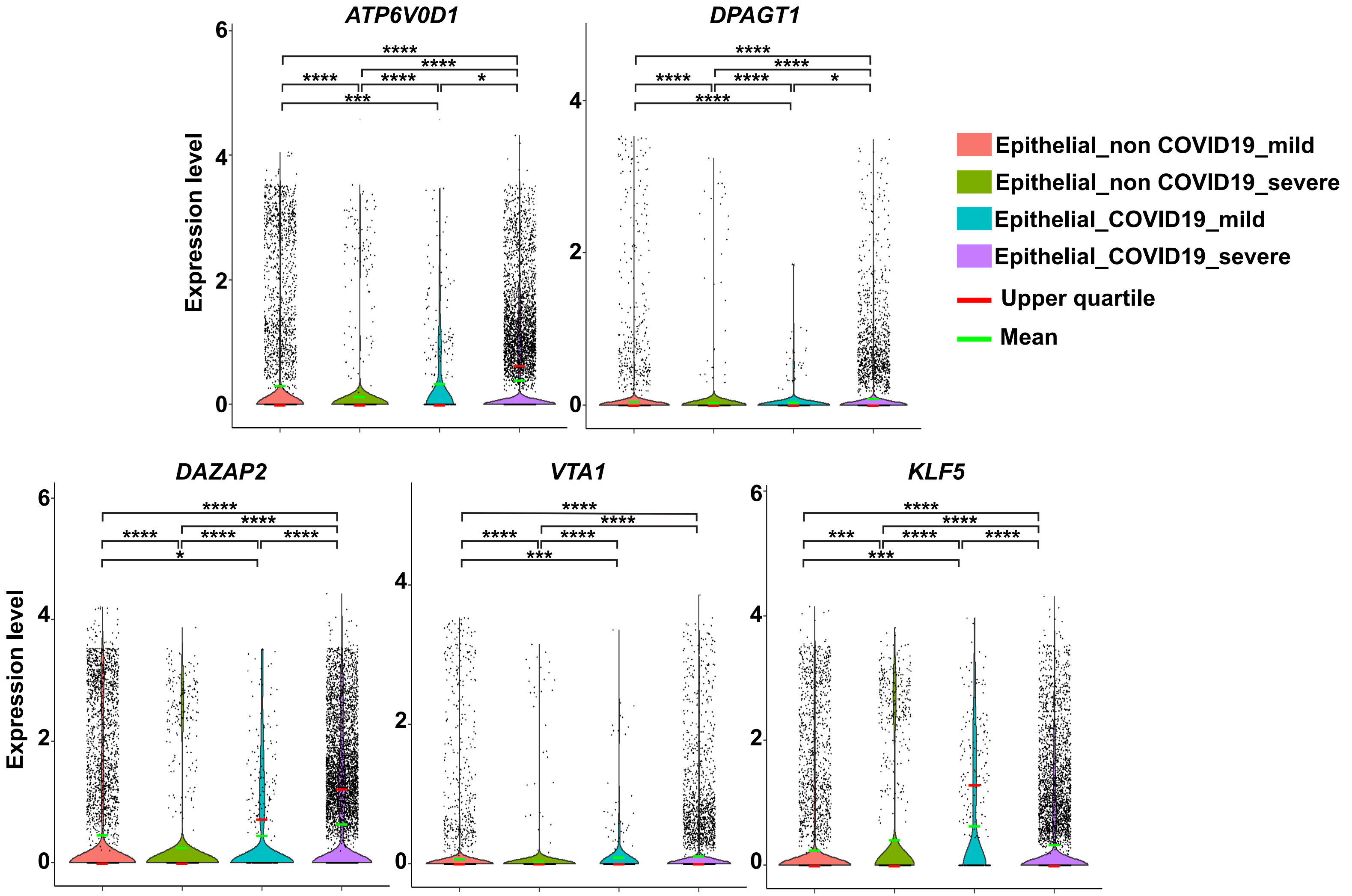
ATP6V0D1
DPAGT1
DAZAP2
VTA1
KLF5
Expression level
Epithelial_non COVID19_mild
Epithelial_non COVID19_severe
Epithelial_COVID19_mild
Epithelial_COVID19_severe
Upper quartile
Mean

**Extended Data Fig. 4. Comparisons of expression levels of identified host factors in lung epithelial cells from pneumonia patients.** The mRNA expression levels of two pro-viral factors (ATP6V0D1and DPAGT1) and three anti-viral factors (DAZAP2, VTA1, and KLF5) in epithelial cells in bronchoalveolar lavage fluids were extracted from published single cell RNA-seq datasets of pneumonia patients. Patients were stratified by their diagnosis (COVID-19 and non-COVID-19) and severity (mild and severe). Log transformed read counts to each host factor in lung epithelial cells from different patient groups were illustrated. The comparisons with statistical significance were indicated. *p<0.05; ***p<0.001; ****p<0.0001.

JHou et.al Extended Data Figure 5

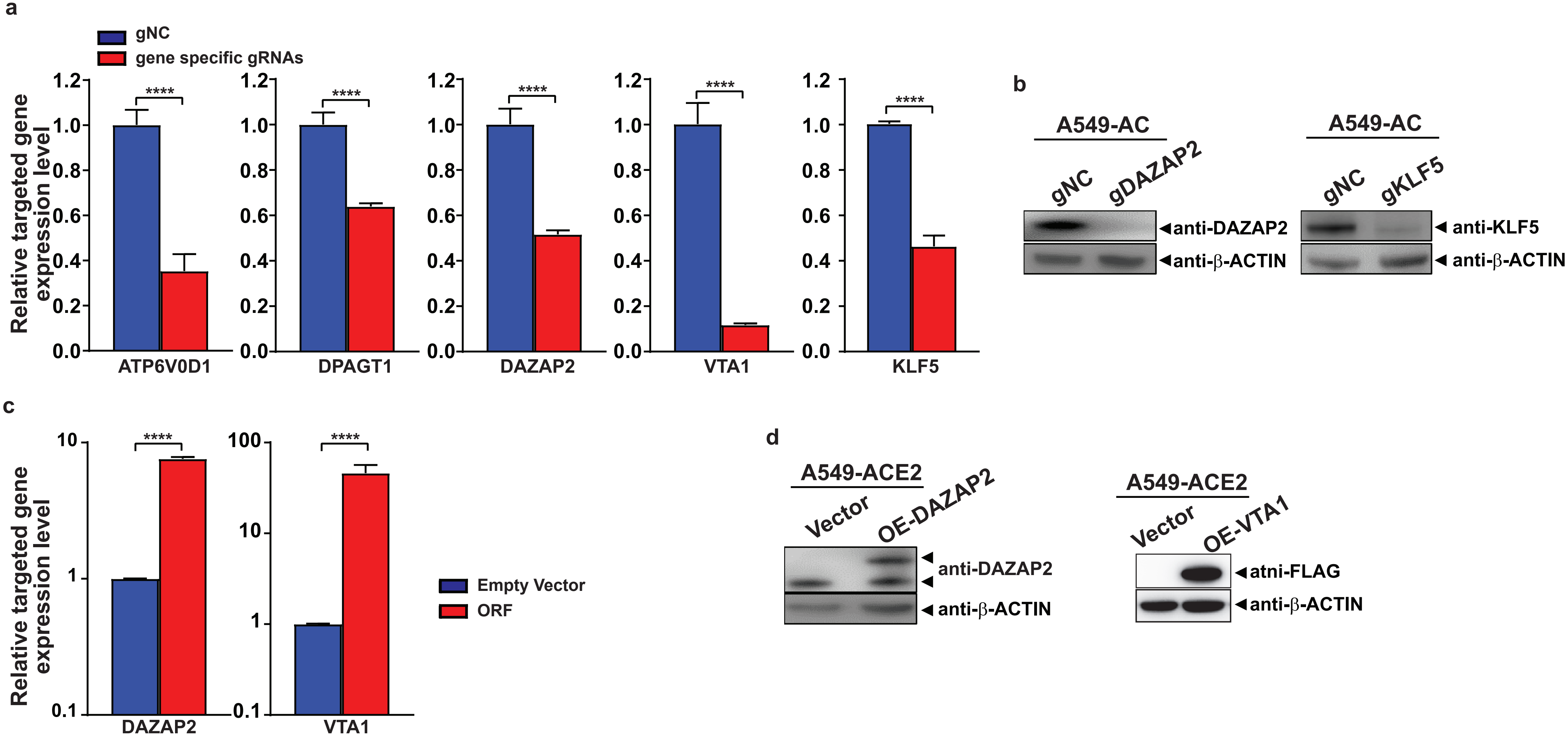

**Extended Data Fig. 5. Successful gene-specific perturbations in A549-AC cells.** (a) Inhibition of mRNA expression of target genes in A549-AC cells by gene-specific gRNAs. (b) Inhibition of expression of target proteins in A549-AC cells by gene-specific gRNAs. (c) Increased mRNA expression of target genes in gene-specific overexpression (OE) A549-AC cells. (d) Increased expression of target protein in gene-specific OE A549-AC cells. The comparisons with statistical significance were indicated. ***$p<0.001$; ****$p<0.0001$.

**Extended Data Table 1. List of pro-viral host factors identified from SARS-CoV-2 dropout screens.**

**Extended Data Table 2. List of anti-viral host factors identified from SARS-CoV-2 dropout screens.**

**Extended Data Table 3. Summary of published genome-wide gRNA SARS-CoV-2 screens for integrative studies.** Publicly available datasets from genome-wide CRISPR screens based on the CPE of SARS-CoV-2 on human epithelial cells were extracted and used to evaluate the performance of identified hits. The evaluation criteria for each dataset were listed.

**Extended Data Table 4. Evaluation of 30 identified host factors in two independent validation experiments.**

**Extended Data Table 5. List of DNA sequences to generate gRNAs targeting 30 identified host factors for validation.**

**Extended Data Table 6. List of primer information to determine mRNA expression by real-time PCR.**
